# Supplementary material for: In-plane hyperbolic polariton tuners in terahertz and long-wave infrared regimes
Source: Nat Commun. 2023 May 11;14:2716. doi: 10.1038/s41467-023-38214-0 (PMC10175486; doi:10.1038/s41467-023-38214-0)
Supplement: Supplementary file 1 — Supplementary Information [file 41467_2023_38214_MOESM1_ESM.pdf]

Supplementary Information

In-plane hyperbolic polariton tuners in terahertz and long-wave infrared regimes

Wuchao Huang[1,†], Thomas G. Folland[4,5,†], Fengsheng Sun[1,†], Zebo Zheng[1,†], Ningsheng Xu[1,2], Qiaoxia Xing[3], Jingyao Jiang[1], Joshua D. Caldwell[4,*], Hugen Yan[3,*], Huanjun Chen[1,*], and Shaozhi Deng[1,*]

[1]State Key Laboratory of Optoelectronic Materials and Technologies, Guangdong Province Key Laboratory of Display Material and Technology, School of Electronics and Information Technology, Sun Yat-sen University, Guangzhou 510275, China
[2]The Frontier Institute of Chip and System, Fudan University, Shanghai 200433, China
[3]State Key Laboratory of Surface Physics, Department of Physics, Key Laboratory of Micro and Nano-Photonic Structures (Ministry of Education), Fudan University, Shanghai 200433, China
[4]Department of Mechanical Engineering, Vanderbilt University, Nashville, TN 37235, USA
[5]Department of Physics and Astronomy, The University of Iowa, Iowa City, IA 52245, USA

*e-mail: chenhj8@mail.sysu.edu.cn; stsdsz@mail.sysu.edu.cn; Josh.caldwell@vanderbilt.edu; hgyan@fudan.edu.cn.

Supplementary Figure 1. Characterizations of the vdW α-MoO_3 flake
Supplementary Figure 2. SEM images of one-dimensional periodic ribbon patterns with different *w* and *θ*
Supplementary Figure 3. Real parts of permittivity of vdW α-MoO_3 along the three crystallographic axes
Supplementary Figure 4. Fano lineshape fittings on reflectance spectra
Supplementary Figure 5. Schematic showing excitation of polariton resonances in the 1D-PRP from far-field
Supplementary Figure 6. Comparison of the energy absorption by an individual ribbon and a typical single ribbon in the 1D ribbon array
Supplementary Figure 7. Simulated optical near-field distributions |*E*| of a typical α-MoO_3 one-dimensional periodic tuner pattern
Supplementary Figure 8. Dependence of resonance frequency on ribbon width in Reststrahlen Band 3
Supplementary Figure 9 Comparison of the polariton lifetimes obtained from far-field and near-field measurements
Supplementary Figure 10 Raman spectroscopic mapping on unpatterned α-MoO_3 flake
Supplementary Figure 11 Raman spectroscopic mapping of α-MoO_3 nanoribbons with different ribbon widths
Supplementary Figure 12. PhP dispersion relations of α-MoO_3 one-dimensional periodic ribbon patterns at different skew angles *θ*
Supplementary Figure 13. The IFCs of PhPs in THz spectral regime

Supplementary Figure 14. Comparison of the FWHM between PNFs and other commercial polarization-insensitive band pass filters
Supplementary Figure 15. Extinction spectra of PNFs in LWIR and THz regimes
Supplementary Figure 16. Extinction ratio and FWHM of the PNFs as a function of the resonance frequency in LWIR and THz regimes

Supplementary Note 1. Fano lineshape fitting on reflectance spectra and extraction of the Q-factors
Supplementary Note 2. Discussion on the excitation mechanism of HPhPs by the 1D-PRP
Supplementary Note 3. Numerical simulation method
Supplementary Note 4. Calculations of Im$r_p(q_{PhPs}, \omega)$
Supplementary Note 5. Extraction and comparison of the polariton lifetimes obtained from far-field and near-field measurements
Supplementary Note 6. Raman mapping on the unpatterned α-MoO_3 and 1D ribbon arrays
Supplementary Note 7. Calculations of PhP dispersion relations $q[\vec{\varepsilon}(\omega), d, \theta]$

Supplementary Table 1. Comparison of the polariton lifetimes measured from the unpatterned α-MoO_3 and 1D ribbon arrays.
Supplementary Table 2. Comparison of the linewidths of phonon modes measured from the unpatterned α-MoO_3 flake and 1D ribbon arrays with long axes parallel to [001] crystallographic direction.
Supplementary Table 3. Parameters used in calculating the relative permittivities of α-MoO_3 in LWIR and THz regimes

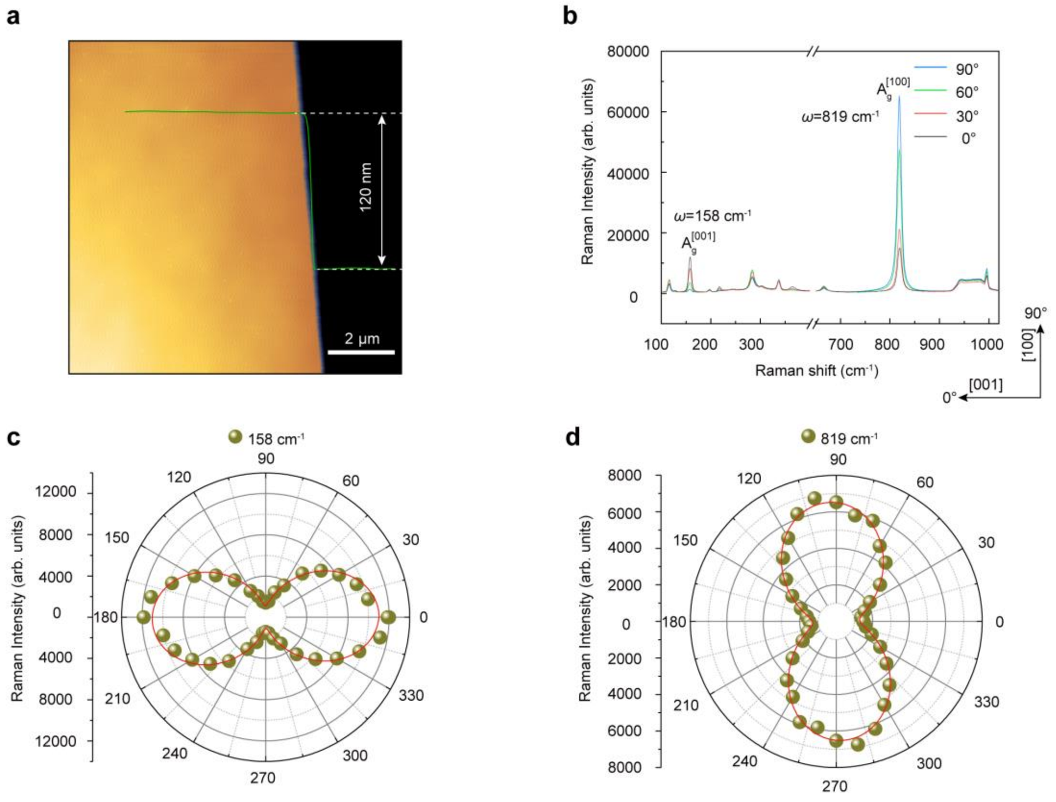

Supplementary Figure 1 | Characterizations of the vdW α-MoO_3 flake. a Atomic force microscope (AFM) topography of the α-MoO_3 flake. **b** Polarization-dependent Raman spectroscopy. The polarization angle is defined as the angle between the excitation polarization and [001] crystallographic axis of the α-MoO_3 flake. **c** and **d** Polar plots of the Raman intensities at the peaks of 158 cm^{-1} (**c**) and 819 cm^{-1} (**d**), respectively.

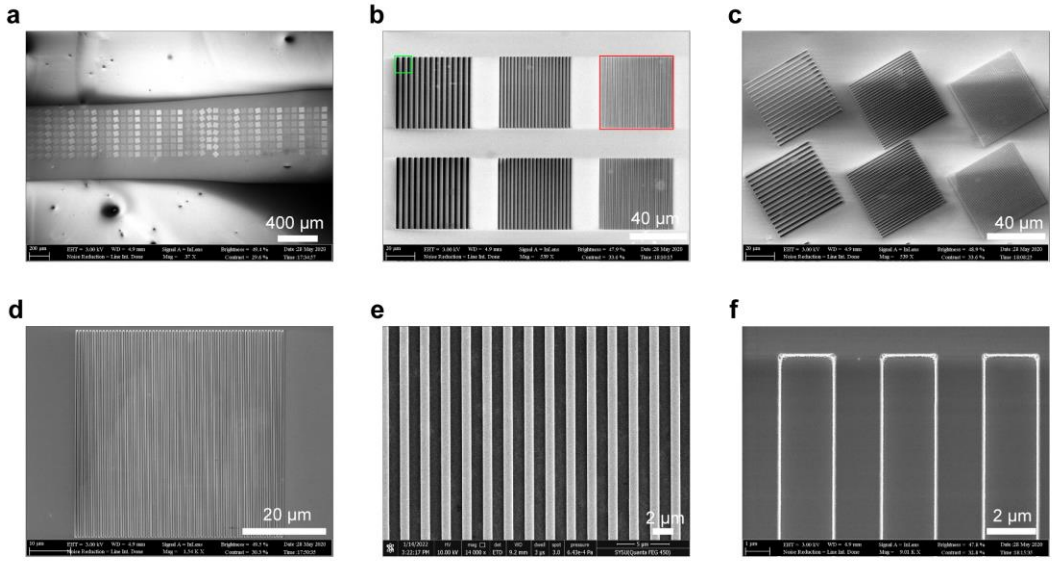

Supplementary Figure 2 | Scanning electron microscope (SEM) images of one-dimensional periodic tuner patterns with different *w* and *θ*. a One-dimensional periodic tuner patterns with different *w* and *θ* fabricated on the same α-MoO_3 flake. **b** Enlarged SEM images of one-dimensional periodic tuner patterns with different *w*. **c** Enlarged SEM images of one-dimensional periodic tuner patterns with different *θ*. **d–f** Enlarged SEM image of the one-dimensional periodic tuner patterns marked with red (**d** and **e**) and green (**f**) colors shown in (**b**).

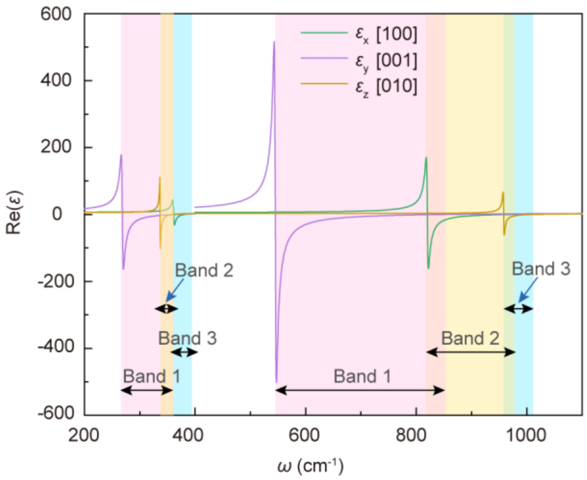

Supplementary Figure 3 | Real parts of permittivity of vdW α-MoO_3 along the three crystallographic axes.

Supplementary Note 1. Fano lineshape fittings on reflectance spectra and extraction of the Q-factors

When a broad resonance interferes with a relatively narrow resonance, the resulting spectrum (absorption, reflectance, transmission, or scattering spectrum) is characterized by an asymmetric non-Lorentzian profile. This phenomenon is well-known as Fano interference or Fano resonance, which was proposed by Ugo Fano[1]. In our study, the reflectance spectra of the pristine α-MoO_3 flake (Fig. 1c) and one-dimensional periodic tuner patterns are asymmetric, suggesting occurrence of the Fano resonances. Specifically, the Fano interferences will occur between the phonon modes (for pristine α-MoO_3 flake) or PhP resonances (for one-dimensional periodic tuner patterns) with narrow linewidths and the background reflectance with a broad linewidth. Therefore, the corresponding reflectance spectra around the resonances (reflectance peaks or valleys) can be fitted using the Fano lineshape[1,2],

$$F(\omega)=\frac{2p}{\pi\Gamma\left(q_f^2+1\right)}\frac{\left[q_f+\frac{2(\omega-\omega_0)}{\Gamma}\right]}{\left[1+\frac{4(\omega-\omega_0)^2}{\Gamma^2}\right]} \tag{S1}$$

where Γ is the linewidth of a specific Fano resonance, p is the amplitude, q_f is the Fano parameter accounting for the lineshape, and ω_0 is the shifted phonon frequency or PhP resonance frequency. Consequently, the Q-factor of the PhP resonance can be evaluated as $Q=\frac{\omega_0}{\Gamma}$.

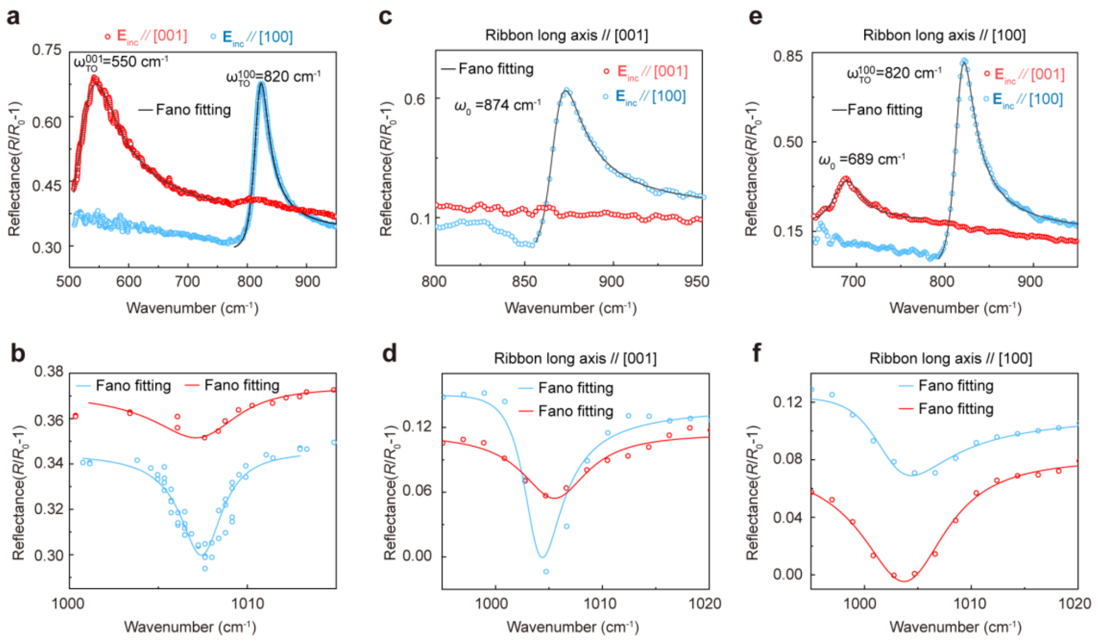

Supplementary Figure 4 | Fano lineshape fittings on reflectance spectra of the pristine α-MoO_3 flake (a and b) and two representative one-dimensional periodic tuner patterns (c–f). The fittings are performed around the reflectance peaks (**a**, **c**, and **e**) and valleys (**b**, **d**, and **f**). The experimental data points are overlaid onto the fitting curves.

Supplementary Note 2. Discussion on the excitation mechanism of HPhPs by the 1D-PRP

To excite the HPhPs from far-field, it is required to compensate the large wavevector mismatch between free-space photons (k_0) and polaritons (q_{PhPs}). In our

study, this can be achieved from two pathways (Fig. S5). Specifically, the first one is formation of Fabry–Pérot resonance (FPR). When the ribbon arrays are illuminated at normal incidence with an electric field pointing perpendicularly to the ribbon longitudinal axis, the ribbon edges will act as subwavelength-scale structures providing evanescent fields with high momentum. The evanescent waves can then hybridize with the optical phonons in α-MoO_3 and excite HPhPs propagating transverse to the ribbons. Once the polaritons are stimulated, they will form standing-wave resonances, *i.e.*, the FPR, by multiple reflections from the ribbon edges under the condition,

$$q_{\mathrm{PhPs}}w+\Phi = m\pi,\ m = 1, 2, 3, \ldots \qquad \text{(S2)}$$

where w is the ribbon width, Φ is the possible phase shift upon reflection at the edges. The second one is that the 1D-PRP acts as a grating structure. The incidence waves will be scattered into guided waves (GWs) propagating transverse to the ribbons if the following condition is satisfied[3],

$$k\Lambda = n2\pi,\ n = 1, 2, 3, \ldots \qquad \text{(S3)}$$

where k and Λ are the wavevector of the GWs and period of the ribbon array, respectively. The GWs can then couple with the α-MoO_3 ribbons and convert into HPhPs with the same wavevectors[4]. Considering that in our study the Λ was deliberately set as $2w$, Eq. S3 can be written as,

$$q_{\mathrm{PhPs}}w = n\pi,\ n = 1, 2, 3, \ldots \qquad \text{(S4)}$$

The Eq. S2 and Eq. S4 are very close to each other except the phase difference Φ in Eq. S2. To ascertain which pathway of the two dominates the excitation of the HPhPs resonances, the energy absorbed by an isolated individual ribbon (labeled as Ribbon 1) and a typical single ribbon (labeled as Ribbon 2) in the 1D-PRP is compared. To that end, the energy consumption and electric field distributions within the Ribbon 1 and Ribbon 2 are calculated. Both ribbons have thicknesses of 200 nm and w of 800 nm. The longitudinal axes of the ribbons are set along [100] crystalline direction. The illumination power is kept the same. As shown in Fig. S6a, the energy absorption in Ribbon 1 and Ribbon 2 is both frequency dependent, with the maxima at ω_{01} = 878 cm^{-1} and ω_{02} = 876 cm^{-1}, respectively. The absorption maximum in Ribbon 2 is an order of magnitude larger than that in Ribbon 1. The ω_{02} coincides with the polariton resonance frequency of the 1D ribbon array extracted from the far-field reflectance spectrum (Fig. S6a, dashed line). Moreover, the electric field distribution inside the Ribbon 1 is much smaller than that in the Ribbon 2 (Fig. S6b). Similar results can also be observed for ribbons orientating along the [001] crystalline direction (Fig. S6c and d). From these analyses it can be deduced that the excitation of HPhPs are originated from the synergy between GWs of the array and FPR in an individual ribbon: the scattering of light at the ribbon edges excite the polariton FPR, while the GWs further couple with and transfer energy to the polariton FPRs.

Due to its periodicity, in our study we define the 1D-PRP as a "1D lattice", giving rise to the reciprocal lattice momentum G. Because the period of the ribbon array is $\Lambda = 2w$, the G then equals to $m\pi/w$.

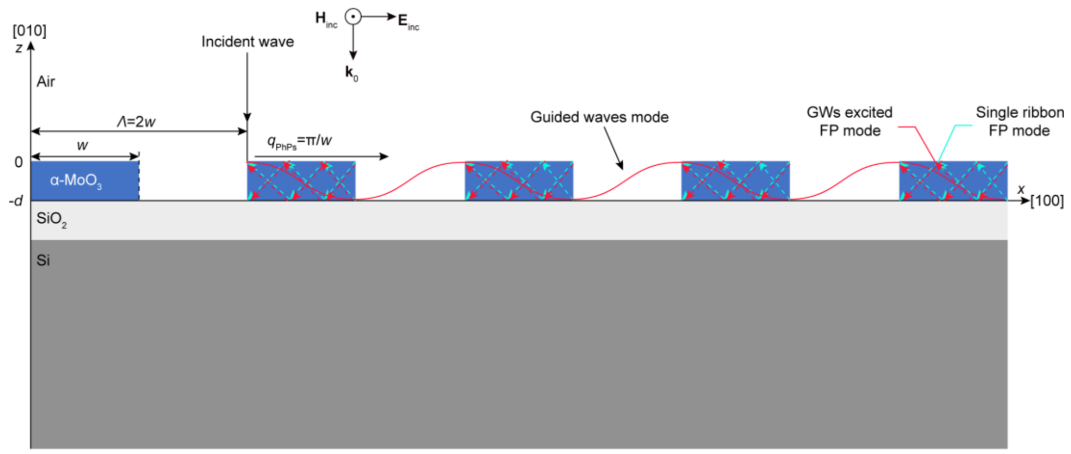

Supplementary Figure 5 | Schematic showing excitation of polariton resonances in the 1D-PRP from far-field. The incident waves will be scattered by the sharp ribbon edges into evanescent waves with large momenta, whereby HPhPs propagating transverse to the ribbons are excited. Fabry−P érot (FP) resonances can then be formed upon the multiple polariton reflections from the ribbon edges. Simultaneously, the 1D-PRPs can also diffract the incident light into guided waves propagating perpendicular to the ribbon long axis, whose wavevectors are much larger than the free-space waves. These guided waves can then couple with and transfer energy to the polariton FP resonances.

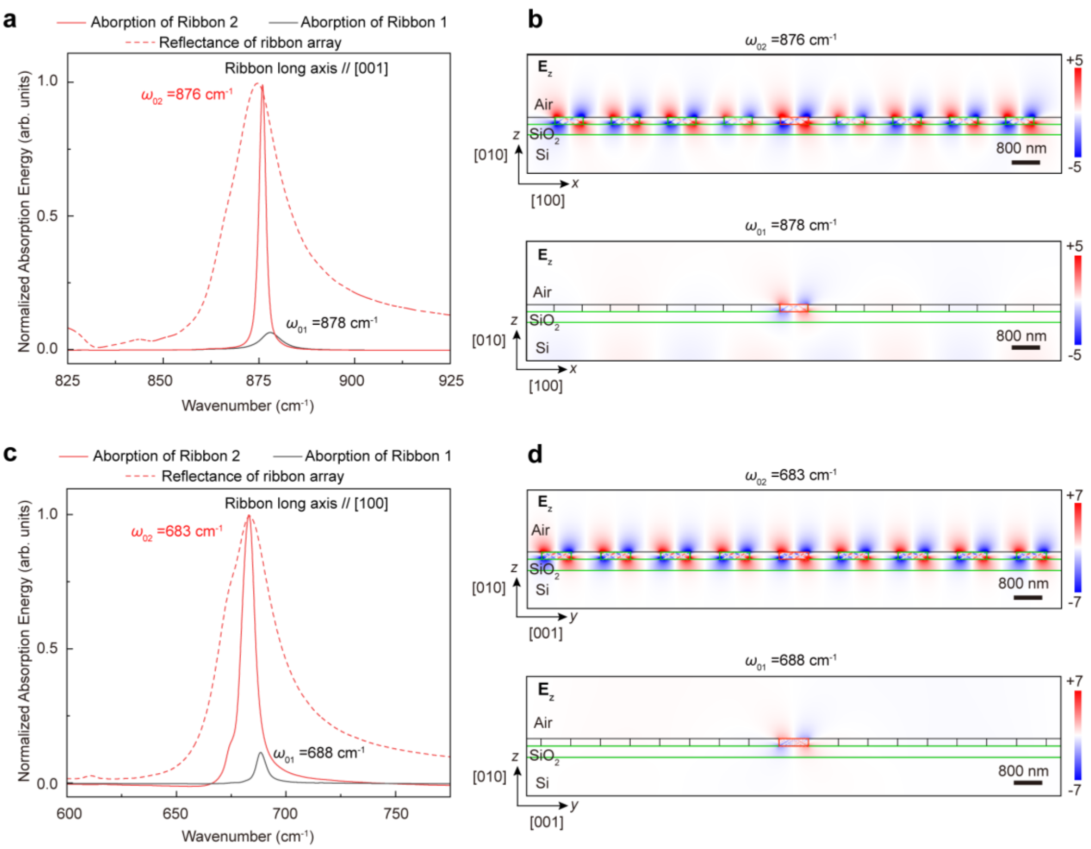

Supplementary Figure 6 | Comparison of the energy absorption by an individual ribbon and a typical single ribbon in the 1D ribbon array. a, c Simulated energy absorption by an individual ribbon (Ribbon 1: black lines) and a typical single ribbon (Ribbon 2: red lines) in the 1D ribbon array. The calculated far-field reflectance

spectra are included for reference (dashed red lines). The longitudinal axes of the ribbons are parallel to [001] (**a**) and [100] (**c**) crystallographic axis of α-MoO_3, respectively. **b**, **d** Optical near-field distributions of Ribbon 1 (lower) and Ribbon 2 (upper). The near-field distributions are drawn on the cross section perpendicular to the ribbon transverse axis, *i.e.*, the *x*–*z* plane for (**b**) and *y*–*z* plane for (**d**).

Supplementary Note 3. Numerical simulations on optical near-field distributions of the α-MoO_3 ribbon

The origin of the resonance peaks in the far-field reflectance spectra of the α-MoO_3 one-dimensional periodic tuner patterns can be unveil by calculating their respective near-field optical distributions. Specifically, for a typical resonance peak, its near-field optical distribution was numerical calculated using the RF module in Comsol, a commercial software capable of solving the Maxwell's equations in the frequency domain. The one-dimensional periodic tuner pattern was placed onto a pristine silicon substrate. A plane wave was utilized as the excitation source, which illuminated the one-dimensional periodic tuner pattern from the air side with an incidence angle of 10 ° with respect to the normal direction. Periodic boundary condition was applied in the simulation. The near-field distribution at a specific resonance peak can be obtained by drawing the modulus of the electric field, |*E*|, were obtained on the cross section perpendicular to the ribbon long axis. The thickness and width of the ribbon were set according to the geometrical parameters obtained with AFM and SEM characterizations, respectively. Permittivity of the silicon substrate was taken from previous reported values[5], while permittivity of α-MoO_3 was modeled by fitting the experimental data using Lorentzian dielectric models[6].

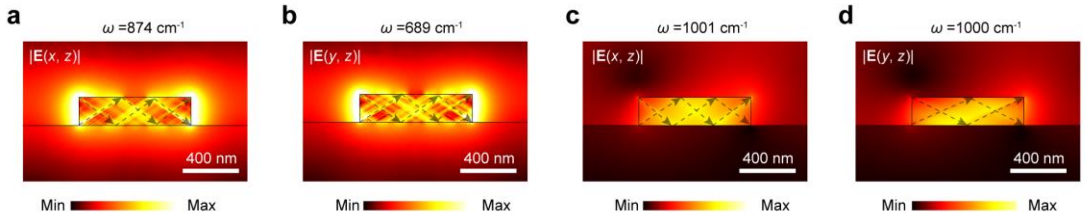

Supplementary Figure 7 | Simulated optical near-field distributions |*E*| of a typical α-MoO_3 one-dimensional periodic tuner pattern at 874 (a), 689 (b), 1001 (c), and 1000 cm^{-1} (d). The near-field distributions are drawn on the cross section perpendicular to the ribbon long axis, *i.e.*, the *x*–*z* plane (**a**, **c**) and *y*–*z* plane (**b**, **d**). Scale bars: 400 nm. The grey dashed arrows indicate the propagation trajectories of the polaritons.

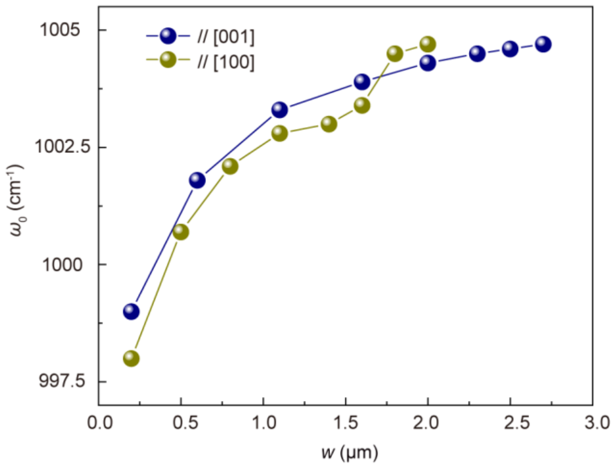

Supplementary Figure 8 | Variation of PhP resonance in Band 3 against the ribbon width. The resonance frequencies are obtained from the spectra shown in Fig. 2a and b in the main text.

Supplementary Note 4. Calculations of Im$r_p(q, \omega)$

The Im$r_p(q, \omega)$ was calculated using a polariton waveguide model developed for vdW crystal[7]. Specifically, the permittivity of the anisotropic α-MoO_3 was modeled as[7],

$$\varepsilon_j = \varepsilon_\infty^j \left(1 + \frac{\omega_{LO}^{j\ 2} - \omega_{TO}^{j\ 2}}{\omega_{TO}^{j\ 2} - \omega^2 - i\omega\Gamma^j}\right), \qquad j = x, y, z \tag{S5}$$

where ε_j denotes the principal components of the permittivity, and the x, y, and z denote the three principal axes of the crystal. The ε_∞^j is the high frequency dielectric constant, the ω_{LO}^j and ω_{TO}^j refer to the LO and TO phonon frequencies, respectively. The parameter Γ^j is the broadening factor of the Lorentzian lineshape, which is determined by the phonon lifetime. All of the parameters used in our calculations are adopted from Ref. 6 (for LWIR regime) and Ref. 5 (for THz regime) with slight modifications to match with the experimental data, which are given in Table S3.

In the principal coordinate system, the dielectric tensor $\vec{\vec{\varepsilon}}$ can be described as,

$$\vec{\vec{\varepsilon}} = \begin{pmatrix} \varepsilon_x & 0 & 0 \\ 0 & \varepsilon_y & 0 \\ 0 & 0 & \varepsilon_z \end{pmatrix} \tag{S6}$$

To study the anisotropic polariton propagation along different directions in the basal plane of the vdW crystal, a coordinate transformation is applied,

$$\vec{\vec{\varepsilon}}' = T\vec{\vec{\varepsilon}}T^{-1} = \begin{pmatrix} \varepsilon_{xx} & \varepsilon_{xy} & \varepsilon_{xz} \\ \varepsilon_{yx} & \varepsilon_{yy} & \varepsilon_{yz} \\ \varepsilon_{zx} & \varepsilon_{zy} & \varepsilon_{zz} \end{pmatrix} = \begin{pmatrix} \varepsilon_y \sin^2\theta + \varepsilon_x \cos^2\theta & (\varepsilon_y - \varepsilon_x)\sin\theta\cos\theta & 0 \\ (\varepsilon_y - \varepsilon_x)\sin\theta\cos\theta & \varepsilon_y \cos^2\theta + \varepsilon_x \sin^2\theta & 0 \\ 0 & 0 & \varepsilon_z \end{pmatrix} \tag{S7}$$

where θ denotes the angle of the polariton propagation direction relative to the x-axis.

In our theoretical model the supported α-MoO_3 is treated as a multilayer structure composed of four layers: air layer ($0 < z$, $j = 0$), α–MoO_3 layer ($-d_1 < z < 0$, $j = 1$), SiO_2 layer ($-d_2 < z < -d_1$, $j = 2$), and Si substrate layer ($z < -d_2$, $j = 3$). For a p-polarized excitation, the in-plane magnetic field in the system are written as,

$$H_y^{(j)}(x,z) = [A_j \exp(-ik_z^{(j)}z) + B_j \exp(ik_z^{(j)}z)]\exp(iqx) \tag{S8}$$

Consequently, we can get,

$$E_x^{(j)}(x,z)=\left(k_z^{(j)}\Big/\omega\varepsilon_t^{(j)}\right)[A_j\exp(-ik_z^{(j)}z)-B_j\exp(ik_z^{(j)}z)]\exp(iqx) \tag{S9}$$

By matching the continuity condition of the electromagnetic fields at the interfaces between adjacent layers (z = 0, $-d_1$, and $-d_2$), we can obtain,

$$\begin{cases} A_0+B_0=A_1+B_1 \\ Q^{(0)}(A_0-B_0)=Q^{(1)}(A_1-B_1) \\ A_1\exp(ik_z^{(2)}d_1)+B_1\exp(-ik_z^{(2)}d_1)=A_2\exp(ik_z^{(2)}d_1)+B_2\exp(-ik_z^{(2)}d_1) \\ Q^{(1)}[A_1\exp(ik_z^{(2)}d_1)-B_1\exp(-ik_z^{(2)}d_1)]=Q^{(2)}[A_2\exp(ik_z^{(2)}d_1)-B_2\exp(-ik_z^{(2)}d_1)] \\ A_2\exp(ik_z^{(2)}d_2)+B_2\exp(-ik_z^{(2)}d_2)=A_3\exp(ik_z^{(3)}d_2) \\ Q^{(2)}[A_2\exp(ik_z^{(2)}d_2)-B_2\exp(-ik_z^{(2)}d_2)]=Q^{(3)}A_3\exp(ik_z^{(3)}d_2) \end{cases} \tag{S10}$$

where $k_z^{(m)}=\sqrt{\varepsilon_t^{(m)}\left(\omega/c\right)^2-\varepsilon_t^{(m)}\Big/\varepsilon_z^{(m)}q^2}$, and $Q^{(m)}=k_z^{(m)}\Big/\varepsilon_t^{(m)}$, ($m$ = 0, 1, 2, 3). In the α-MoO_3 layer, $\varepsilon_t=\varepsilon_y\sin^2\theta+\varepsilon_x\cos^2\theta$. The relation between the coefficients can be obtained as,

$$\begin{cases} A_0=A_1\dfrac{Q^{(0)}+Q^{(1)}}{2Q^{(0)}}+B_1\dfrac{Q^{(0)}-Q^{(1)}}{2Q^{(0)}}, \\ A_1=A_2\dfrac{Q^{(1)}+Q^{(2)}}{2Q^{(1)}}\exp[-i(k_z^{(1)}-k_z^{(2)})d_1]+B_2\dfrac{Q^{(1)}-Q^{(2)}}{2Q^{(1)}}\exp[-i(k_z^{(1)}+k_z^{(2)})d_1], \\ A_2=1, \\ A_3=\dfrac{2Q^{(2)}}{Q^{(2)}+Q^{(3)}}\exp[i(k_z^{(2)}-k_z^{(3)})d_2], \\ B_0=A_1\dfrac{Q^{(0)}-Q^{(1)}}{2Q^{(0)}}+B_1\dfrac{Q^{(0)}+Q^{(1)}}{2Q^{(0)}}, \\ B_1=A_2\dfrac{Q^{(1)}-Q^{(2)}}{2Q^{(1)}}\exp[i(k_z^{(1)}+k_z^{(2)})d_1]+B_2\dfrac{Q^{(1)}+Q^{(2)}}{2Q^{(1)}}\exp[i(k_z^{(1)}-k_z^{(2)})d_1], \\ B_2=\dfrac{Q^{(2)}-Q^{(3)}}{Q^{(2)}+Q^{(3)}}\exp(2ik_z^{(2)}d_2), \\ B_3=0. \end{cases} \tag{S11}$$

The complex reflectivity r_p of the air/α-MoO_3/SiO_2/Si multilayer structure is then given by,

$$r_p(q,\omega)=-\frac{B_0}{A_0}=-\frac{A_1\left[Q^{(0)}-Q^{(1)}\right]+B_1\left[Q^{(0)}+Q^{(1)}\right]}{A_1\left[Q^{(0)}+Q^{(1)}\right]+B_1\left[Q^{(0)}-Q^{(1)}\right]} \tag{S12}$$

where q represents polariton wave vector away from the x-axis by an angle θ. One should note that by plotting the Im$r_p(q, \omega)$, only the real part of q was considered.

Supplementary Note 5. Extraction and comparison of the polariton lifetimes obtained from far-field and near-field measurements

We extracted the lifetime of HPhPs in the 1D-PRPs by fitting the reflection spectra with the Fano line-shape function (Eq. S1 and Fig. S9a). The lifetime τ at a resonance frequency ω_0 can be derived as $\tau=2\hbar/\Gamma$. As shown in Fig. S9c, the

polariton lifetime of the 1D-PRPs ranges from 0.2 ps to 3.0 ps for Reststrahlen Band 1 to Band 3. We also performed near-field nano-imaging measurements on the same α-MoO_3 flake in the unpatterned region. A scattering type scanning near-field optical microscope was employed for the near-field characterizations (neaSNOM, neaspec GmbH)[6,7]. It should be noted that due to the limitation of the quantum cascade lasers integrated into our s-SNOM system, the near-field measurements can only be conducted in the frequency range of 890 to 1250 cm^{-1} (Band 2 and 3). In addition, to make a direct comparison, the excitation frequency of the s-SNOM was set according to the resonance frequency of the 1D-PRPs measured in the far-field. Specifically, for an 1D-PRP with the ribbon longitudinal axis pointing along [100] ([001]) direction, the resonance frequency was measured from far-field as ω_0. The same frequency ω_0 was then employed as the excitation frequency for the near-field measurements. Consequently, clear interference fringes can be observed along the [001] ([100]) crystallographic direction (Fig. S9b). According to the line profiles extracted from the near-field interference fringes (Fig. S9b, insets), it is able to obtain the propagation length of the HPhPs upon an excitation frequency of ω_0. A typical line profile along [100] crystallographic direction of α-MoO_3 can be fitted using the equation[8],

$$y = Ax^{-0.5} e^{-\frac{x}{t_0}} \sin\left(\pi \frac{x - x_c}{w}\right), \quad A > 0, \quad w > 0, \quad t_0 > 0. \tag{S13}$$

where t_0 denotes the polariton propagation length. The group velocity v_g of the HPhPs in α-MoO_3, which is defined as $v_g = \partial\omega/\partial k$, can be extracted from the polariton dispersion relation calculated using the analytical model (see Note S7 below). Afterwards, the lifetime of the HPhPs in the unpatterned α-MoO_3 can be calculated as $\tau = L/v_g$.

The comparison of the polariton lifetimes obtained from far-field and near-field measurements is shown in Fig. S9c and Table S1.

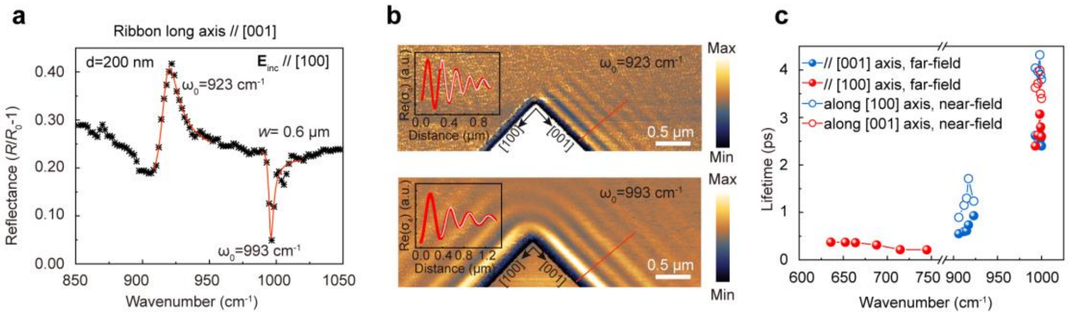

Supplementary Figure 9 | Comparison of the polariton lifetimes obtained from far-field and near-field measurements. a Fano lineshape fitting on a typical reflectance spectrum of the α-MoO_3 1D-PRPs. The fitting was performed around the reflectance peaks and valleys. The experimental data points (symbols) are overlaid onto the fitting curve. **b** Experimental near-field images of unpatterned α-MoO_3 at the resonance frequencies of ω_0 = 923 cm^{-1} (upper panel) and 993 cm^{-1} (lower panel). Scale bars: 0.5 µm. Insets: typical near-field line profiles along the [100] crystallographic direction (solid red lines) of α-MoO_3. **c** Comparison of the polariton lifetimes of the unpatterned α-MoO_3 (opened symbols) and ribbon arrays (solid symbols) at various resonance frequencies. The thickness of the α-MoO_3 is d = 200 nm. The solid spheres are extracted from the far-field spectroscopic measurements, while the open spheres are results from near-field characterizations. For far-field measurements, the long axes of the ribbons are paralleled to the [100] (red) and [001] (blue) crystallographic directions, respectively. For near-field measurements, the line

profiles are analyzed along [001] (red) and [100] (blue) crystallographic directions, respectively.

Supplementary Table 1. Comparison of the polariton lifetimes measured from the unpatterned α-MoO_3 and 1D ribbon arrays.

Frequencies (cm^{-1})	A: lifetime from unpatterned α-MoO_3 (ps)	B: lifetime from 1D ribbon arrays (ps)	Difference: (A–B)/A
	**Line Profile of near-field images along [001]**	**Ribbon longitudinal axis // [100]**	
636	--	0.37	--
652.5	--	0.37	--
664	--	0.36	--
688	--	0.31	--
714.8	--	0.21	--
745	--	0.20	--
993	3.62	2.40	34%
996	3.71	2.63	29%
998	3.99	3.00	23%
999	3.50	2.80	20%
1000	3.40	2.60	23%
**Frequencies (cm^{-1})**	**C: lifetime from unpatterned α-MoO_3 (ps)**	**D: lifetime from 1D ribbon arrays (ps)**	**Difference: (C–D)/C**
	**Line Profile of near-field images along [100]**	**Ribbon longitudinal axis // [001]**	
906	0.89	0.55	38%
912	1.15	0.59	48%
915	1.31	0.61	53%
917	1.71	0.73	57%
923	1.23	0.93	25%
993	4.03	2.62	35%
996	3.96	2.46	38%
998	4.32	2.65	38%
999	3.91	2.55	34%
1000	3.83	2.41	37%

Supplementary Note 6. Raman mapping on the unpatterned α-MoO_3 and 1D ribbon arrays

The linewidth γ of the phonon modes can be obtained by performing spatially-resolved two-dimensional (2D) Raman mapping on both unpatterned α-MoO_3 and 1D ribbon arrays using a confocal Raman spectrometer (Renishaw inVia Reflex). A typical Raman spectrum of the unpatterned α-MoO_3 flake is shown in Fig. S10a. The linewidths of the phonon modes at 820 cm^{-1} and 995 cm^{-1}, which originate from the vibrational modes along the [100] and [010] crystallographic directions, respectively, can be readily obtained by fitting the spectrum with a Lorentzian lineshape. In the Raman mapping measurement, the Raman spectra at each position were obtained by scanning the sample under the laser spot. The 2D pseudo-colored images shown in Fig. S10b and S10c visualize the spatial distribution of the phonon linewidths over the unpatterned region. The phonon linewidth is rather homogeneous, with 8.0 $cm^{-1} \leq \gamma \leq$ 9.0 cm^{-1} for the mode at 820 cm^{-1} and 3.0 $cm^{-1} \leq \gamma \leq$ 3.5 cm^{-1} for that at 995 cm^{-1}, respectively.

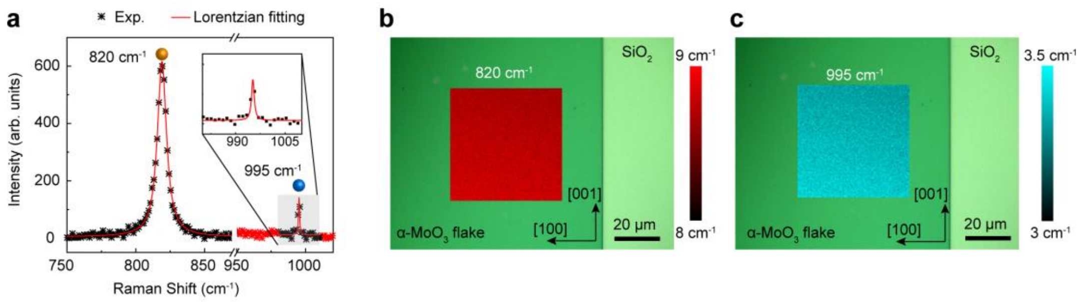

Supplementary Figure 10 | Raman spectroscopic mapping on unpatterned α-MoO_3 flake. a Typical Raman spectrum of the unpatterned α-MoO_3 flake. Inset: enlarged spectrum from 980 cm^{-1} to 1010 cm^{-1}. The symbols correspond to experimental data while the red lines are Lorentzian lineshape fittings on the experimental spectra. **b**, **c** Two-dimensional Raman mapping on the unpatterned α-MoO_3 flake. The mappings correspond to linewidths of the phonon modes at 820 cm^{-1} (**b**) and 995 cm^{-1} (**c**), respectively. The polarization of the incident light is parallel to the [100] crystallographic direction. The mapping was conducted over an area of 50 μm×50 μm (red and blue regions in (b) and (c), respectively). The scale bars are 20 μm.

Afterwards, we conducted 2D Raman mapping on α-MoO_3 nanoribbons with widths of w = 1.0, 2.0, 2.5, and 3.0 μm. The mapping was performed to enclose the ribbon edges, as shown in Fig. S11 (pseudo-colored regions). Compared to the unpatterned flake, the overall linewidths of phonon modes at 820 cm^{-1} and 995 cm^{-1} in these ribbons increased by 4.6% to 14.1% (Table S2). Notably, the ribbon edges exhibited larger linewidths than the center of the ribbon (Fig. S11a−S11d) due to defects and impurities introduced during fabrication processes. Therefore, the linewidths of phonon modes are not uniform across the nanoribbon. Additionally, the phonon linewidths increased significantly in the nanoribbon region, particularly at the ribbon edges, compared to the unpatterned region. This broadening contributes to the reduced lifetime of the HPhPs in the nanoribbons.

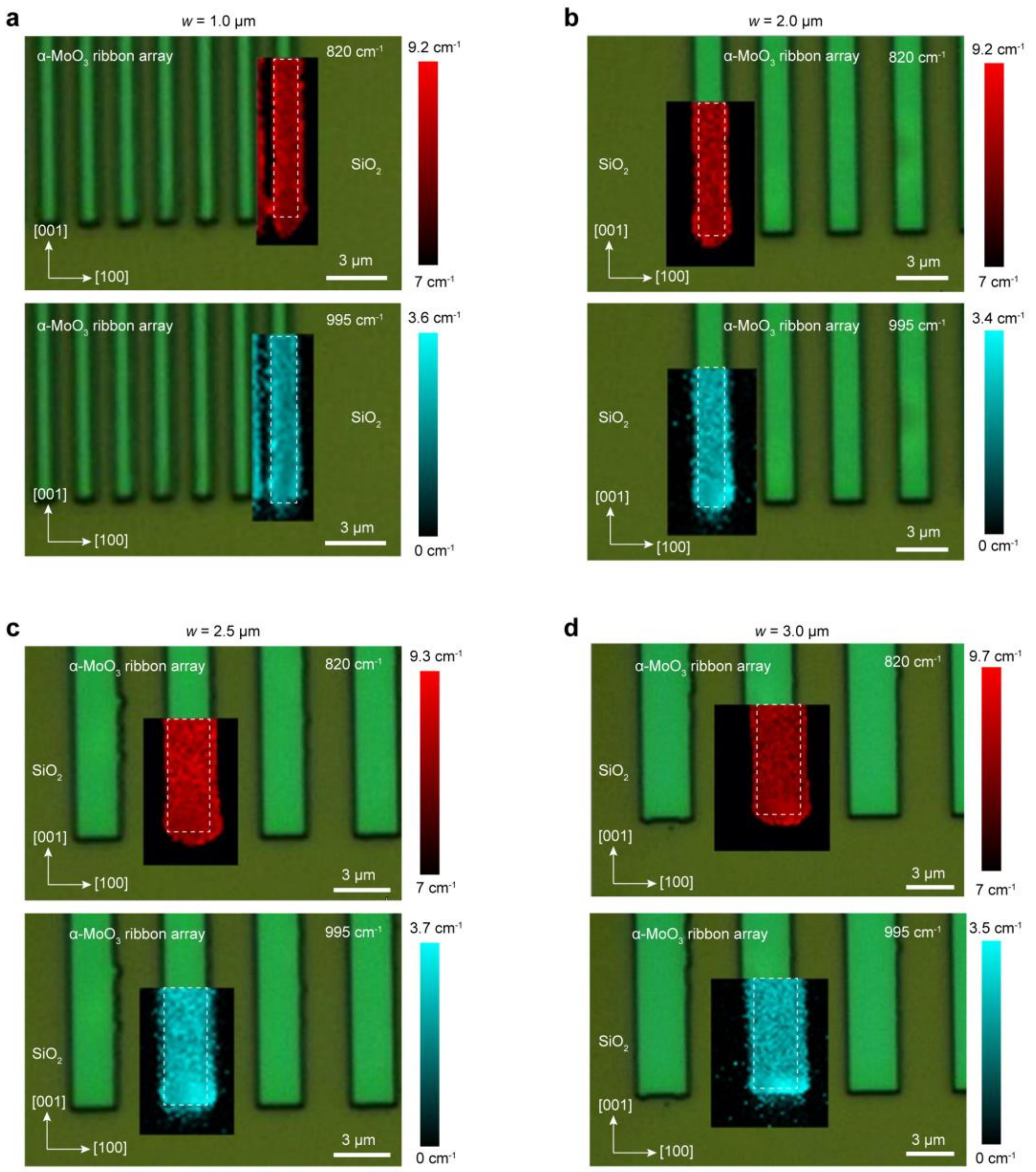

Supplementary Figure 11 | Raman spectroscopic mapping of α-MoO_3 nanoribbons with widths of 1.0 μm (a), 2.0 μm (b), 2.5 μm (c), and 3.0 μm (d). The mappings correspond to linewidths of the phonon modes at 820 cm^{-1} and 995 cm^{-1}, respectively. The polarization of the incident light is parallel to the [100] crystallographic direction. The mapping was conducted to enclose a typical ribbon (pseudo-colored regions shown in (a) to (d)). The scale bars are 3 μm.

Supplementary Table 2. Comparison of the linewidths of phonon modes measured from the unpatterned α-MoO_3 flake and 1D ribbon arrays with long axes parallel to [001] crystallographic direction.

Raman Shift (cm^{-1})	A: linewidth from unpatterned α-MoO_3 (cm^{-1})	*B: linewidth from 1D ribbon arrays (cm^{-1})				Difference: (B–A)/A			
		Width of Ribbon (μm)				Width of Ribbon (μm)			
		1.0	2.0	2.5	3.0	1.0	2.0	2.5	3.0

820	8.5	9.2	9.2	9.3	9.7	8.2%	8.2%	9.4%	14.1%
995	3.25	3.6	3.4	3.7	3.5	10.7%	4.6%	13.8%	7.6%

*Maximum value from the Raman mapping on a specific nanoribbon shown in Fig. S11 is used for the linewidths of the phonon modes.

Supplementary Note S7. Calculations of PhP dispersion relations $q[\vec{\varepsilon}(\omega),d,\theta]$

The solution of polariton electric field has the form as,

$$\vec{E}(\vec{r},t)=\vec{E}(\vec{r})\exp[i(\vec{q}\cdot\vec{r}-\omega t)] \quad \text{(S14)}$$

where $\vec{q}$ represents wave vectors of the PhP. In α-MoO_3 one can only consider the transverse magnetic modes (TM: E_x, H_y, E_z)[7]. Therefore, solutions of the Maxwell equations should satisfy,

$$\begin{aligned}
&\frac{\partial E_x}{\partial z}-\frac{\partial E_z}{\partial x}=-i\omega\mu_0 H_y\\
&\frac{\partial H_z}{\partial y}-\frac{\partial H_y}{\partial z}=i\omega\varepsilon_0\varepsilon_x E_x\\
&\frac{\partial H_z}{\partial x}-\frac{\partial H_x}{\partial y}=i\omega\varepsilon_0\varepsilon_z E_z
\end{aligned} \quad \text{(S15)}$$

To simplify the model, the vdW crystal is treated as a 2D infinite waveguide of thickness d sandwiched between two semi-infinite plates, which are the substrate and cover layer, respectively. Because the PhPs are confined electromagnetic modes, the solution should have the form as,

$$H_y=\begin{cases}A\exp(-\alpha_c z), & z\geq 0\\ A\cos(k_z z)+B\sin(k_z z), & -d\leq z<0\\ [A\cos(k_z d)-B\sin(k_z d)]\exp[\alpha_s(z+d)], & z<-d\end{cases} \quad \text{(S16)}$$

where $\alpha_c=\sqrt{q^2-k_0{}^2\varepsilon_c}$, $\alpha_s=\sqrt{q^2-k_0{}^2\varepsilon_c}$, and $k_z=\sqrt{k_0{}^2\varepsilon_x-(\varepsilon_x/\varepsilon_z)q^2}$.; $k_0 = 2\pi/\lambda_0$ is the free space wave vector.; q is the propagation wave vector of PhPs along a specific direction. Parameters ε_c, ε_x, and ε_s are the permittivities of the cover layer (z > 0), waveguide layer ($-d \leq z \leq 0$), and substrate ($z < -d$), respectively. The electric field E_x can then be obtained as,

$$E_x=\begin{cases}-\dfrac{i\alpha_c}{\omega\varepsilon_0\varepsilon_c}A\exp(-\alpha_c z), & z\geq 0\\ -\dfrac{ik_z}{\omega\varepsilon_0\varepsilon_x}[A\sin(k_z z)-B\cos(k_z z)], & -d\leq z<0\\ \dfrac{i\alpha_c}{\omega\varepsilon_0\varepsilon_c}[A\cos(k_z d)-B\sin(k_z d)]\exp[\alpha_s(z+d)], & z<-d\end{cases} \quad \text{(S17)}$$

where ω is the excitation frequency. According to the continuity of tangential components of electric fields at the interfaces z = 0 and $z = -d$, the following relation can be obtained,

$$\begin{pmatrix}\dfrac{\alpha_c}{\varepsilon_c} & \dfrac{k_z}{\varepsilon_x}\\ \dfrac{k_z}{\varepsilon_x}\sin(k_z d)-\dfrac{\alpha_s}{\varepsilon_s}\cos(k_z d) & \dfrac{k_z}{\varepsilon_x}\cos(k_z d)+\dfrac{\alpha_s}{\varepsilon_s}\sin(k_z d)\end{pmatrix}\begin{pmatrix}A\\B\end{pmatrix}=0 \quad \text{(S18)}$$

To have non-trivial solution of A and B, the determinant of coefficient should be

zero. As a result, the expression of PhPs dispersion relation $q[\vec{\varepsilon}(\omega), d, \theta]$ can be obtained as[7],

$$\sqrt{\frac{\varepsilon_t}{\varepsilon_z}}\sqrt{k_0^2\varepsilon_z - q^2}\,d = \arctan\left(\frac{\sqrt{\varepsilon_t\varepsilon_z}}{\varepsilon_c}\frac{\sqrt{q^2 - k_0^2\varepsilon_c}}{\sqrt{k_0^2\varepsilon_z - q^2}}\right) + \arctan\left(\frac{\sqrt{\varepsilon_t\varepsilon_z}}{\varepsilon_s}\frac{\sqrt{q^2 - k_0^2\varepsilon_s}}{\sqrt{k_0^2\varepsilon_z - q^2}}\right) + M\pi \qquad \text{S(19)}$$

where $\varepsilon_t = \varepsilon_y \sin^2\theta + \varepsilon_x \cos^2\theta$, with θ denotes the angle of the propagation direction relative to the [100] crystal direction in the basal plane of the α-MoO_3, and M = 0, 1, 2, … represents order of the different TM modes. One should note that by plotting the dispersion relation, only the real part of q was considered.

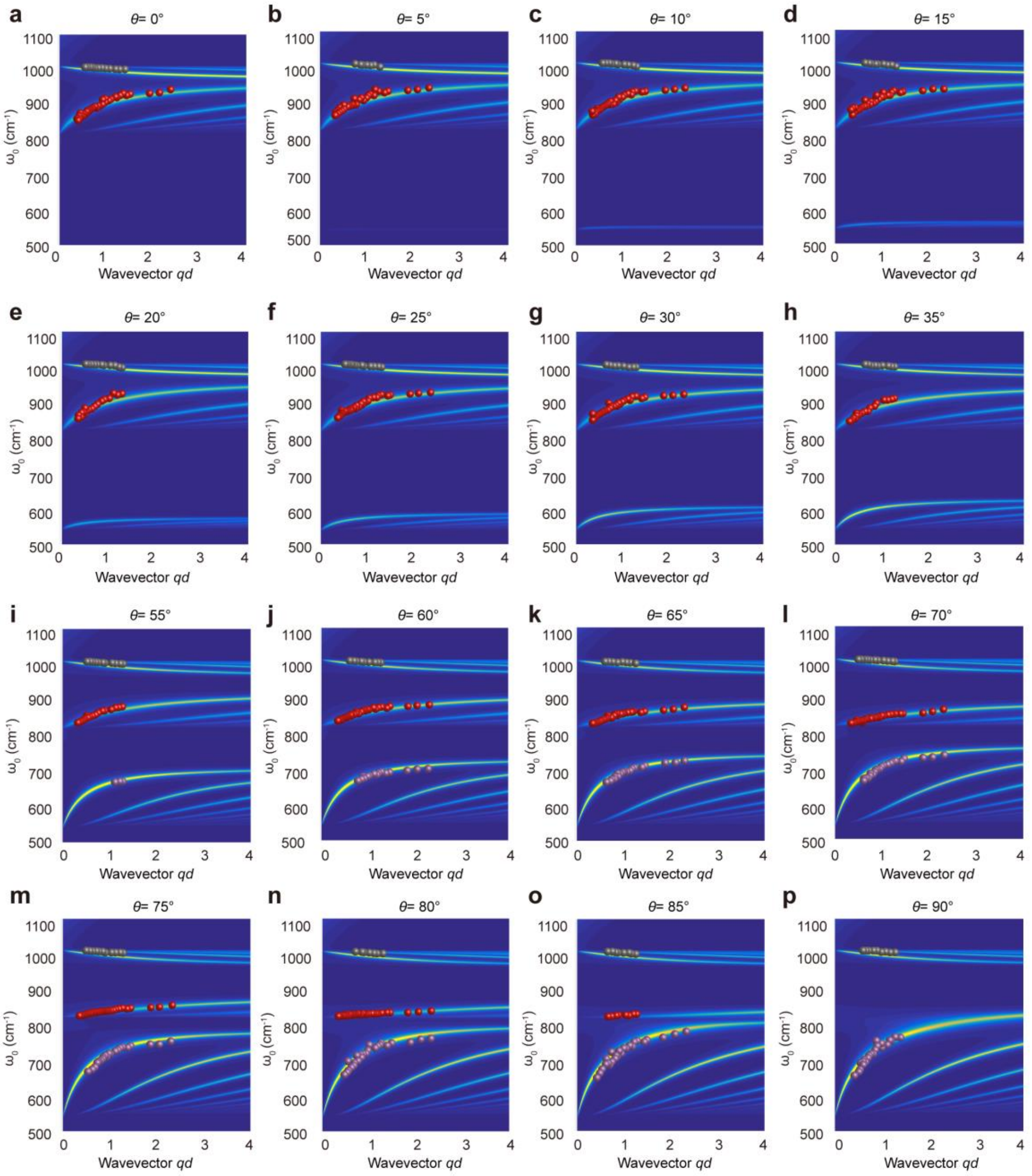

Supplementary Figure 12 | PhP dispersion relations of α-MoO_3 one-dimensional periodic tuner patterns at different skew angles *θ*. The color spheres indicate experimental data extracted from reflectance spectra at different *w*. The false color plots represent the calculated Im$r_p(q_{PhPs}, \omega)$ of the air/α-MoO_3/SiO_2/Si multilayered

structure. The polariton wave vector is normalized by the thickness of the α-MoO_3 flake.

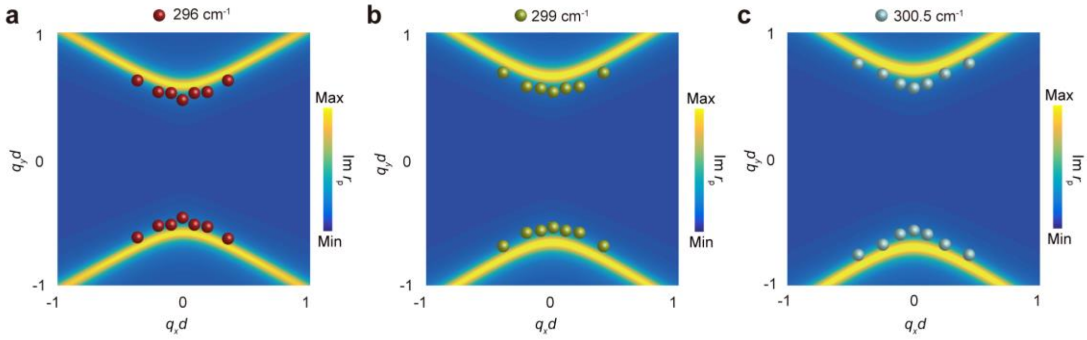

Supplementary Figure 13 | The IFCs of PhPs in THz spectral regime. a–c Polariton IFCs at various frequencies in Band 1. The false color plots represent the Im$r_p(\omega, q_x, q_y)$. The colored spheres in the first quadrant represent the experimental resonance peaks at 296 ± 0.8, 299 ± 0.7, 300.5 ± 0.5 cm^{-1}. Spheres in in other quadrants are duplicated according to the symmetry of the measurement scheme and the α-MoO_3 crystal. The wave vectors in (**a–c**) are normalized by the thickness of the α-MoO_3 flake.

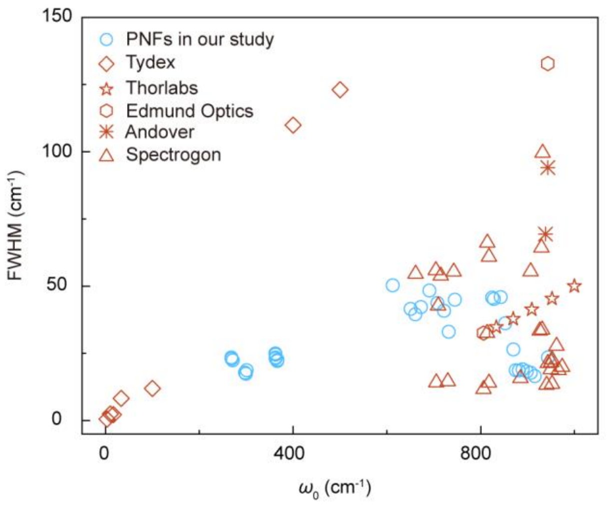

Supplementary Figure 14 | Comparison of the FWHM between PNFs and other commercial band pass filters.

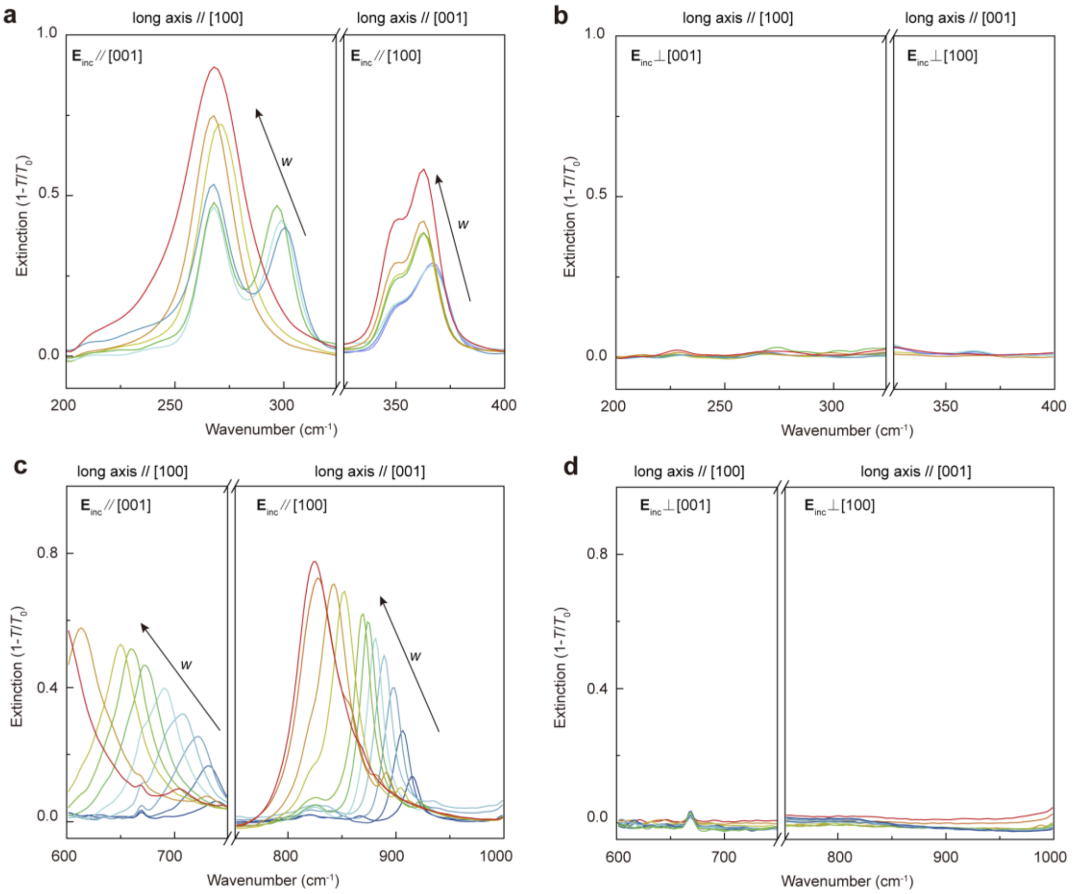

Supplementary Figure 15 | Extinction spectra of PNFs in LWIR and THz regimes. **a**, **b** Polarized extinction spectra of THz PNFs with different *w*. **c**, **d** Polarized extinction spectra of FWIR PNFs with different *w*. The long axes of the one-dimensional periodic tuner patterns are parallel to [100] (left panels) and [001] (right panels) crystallographic directions, respectively. The black arrows indicate the increments of *w*.

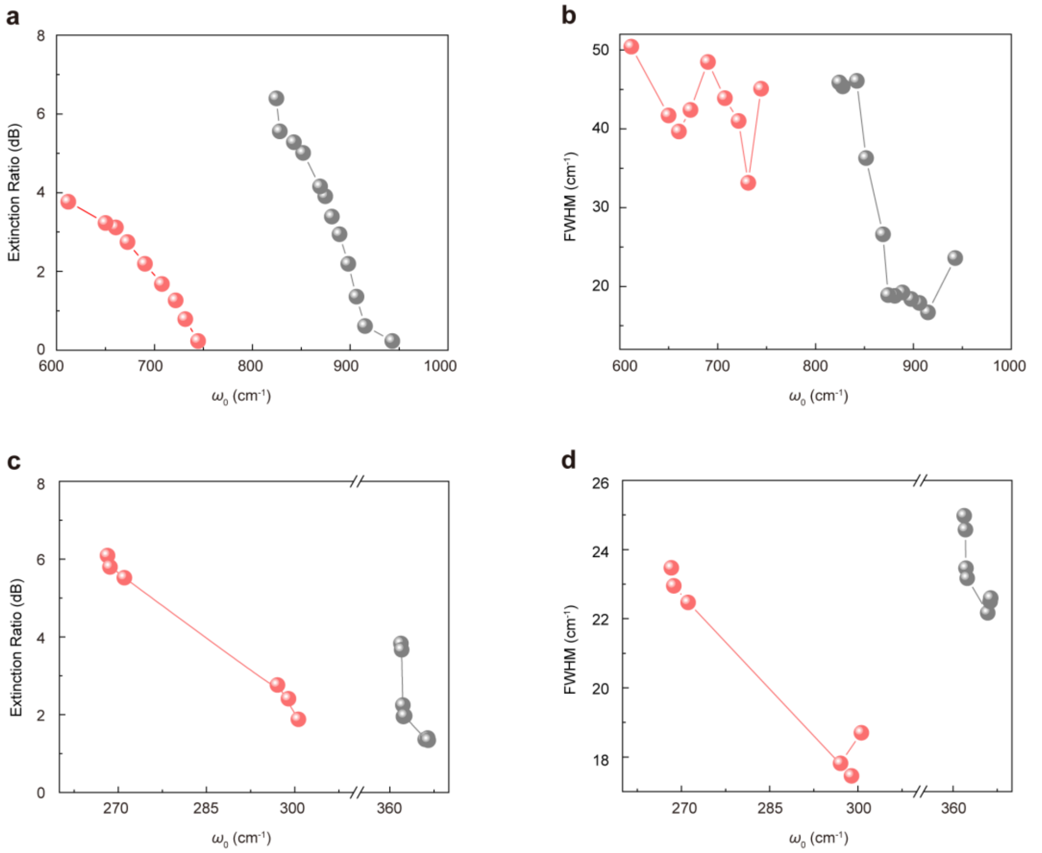

Supplementary Figure 16 | Extinction ratio (**a**, **c**) and FWHM (**b**, **d**) of the PNFs as a function of the resonance frequency ω_0 in LWIR (**a**, **b**) and THz (**c**, **d**) regimes. The widths/periods of the tuner patterns change from 0.4 µm/0.8 µm to 8.0 µm/16.0 µm. The long axes of the ribbons are parallel to [100] (red spheres) and [001] (grey spheres) crystallographic directions, respectively.

Supplementary Table 3. Parameters used in calculating the relative permittivities of α-MoO_3 in LWIR and THz regimes.

**α-MoO_3 (LWIR)**	*x* [100]	*y* [001]	*z* [010]
ε_∞	4.0	5.2	2.4
ω_{LO} /cm^{-1}	972	851	1004
ω_{TO} /cm^{-1}	820	545	958
Γ /cm^{-1}	4	4	2
**α-MoO_3 (THz)**	*x* [100]	*y* [010]	*z* [001]
ε_∞	5.78	4.47	6.07
ω_{LO} /cm^{-1}	385	363	367
ω_{TO} /cm^{-1}	362.4	337	268.4
Γ /cm^{-1}	3	1	4

Supplementary References

1. Fano, U. Effects of configuration ineraction on intensities and phase shifts. *Phys. Rev.* **124**, 1866–1878 (1961).

2. Yan, H. *et al*. Tunable phonon-induced transparency in bilayer graphene nanoribbons. *Nano Lett*. **14**, 4581–4586 (2014).
3. Wang, S. *et al*. Theory and applications of guided-mode resonance filters. *Appl. Opt*. **32**, 2606–2613 (1993).
4. Gao, W. *et al*. Excitation of plasmonic waves in graphene by guided-mode resonances. *ACS Nano* **6**, 7806–7813 (2012).
5. De Oliveira, T. V. *et al*. Nanoscale-confined terahertz polaritons in a van der Waals crystal. *Adv. Mater*. **33**, 2005777 (2021).
6. Zheng, Z. *et al*. Highly confined and tunable hyperbolic phonon polaritons in van der Waals semiconducting transition metal oxides. *Adv. Mater*. **30**, 1705318 (2018).
7. Sun, F. *et al*. Polariton waveguide modes in two-dimensional van der Waals crystals: an analytical model and correlative nano-imaging. *Nanoscale* **13**, 4845–4854 (2021).
8. Ma, W. *et al*. In-plane anisotropic and ultra-low-loss polaritons in a natural van der Waals crystal. *Nature* **562**, 557–562 (2018).
